# Coronary artery vasculitis: a review of current literature

**DOI:** 10.1186/s12872-020-01813-6

**Published:** 2021-01-06

**Authors:** Shaun Khanna, Kartheek Garikapati, Daniel S. L. Goh, Kenneth Cho, Phillip Lo, Mohan V. Bhojaraja, Surjit Tarafdar

**Affiliations:** 1 Department of Medicine, Blacktown Hospital, 18 Blacktown Road, Blacktown, NSW 2148 Australia; 2 Department of Medicine, KMC, Manipal, Karnartaka 576104 India; 3 Faculty of Medicine, Western Sydney University, Sydney, NSW Australia

**Keywords:** Coronary artery vasculitis, Kawasaki’s disease, Takayasu’s arteritis, Giant cell arteritis, Polyarteritis nodosa

## Abstract

Cardiac vasculitis is recognized as a heterogeneous disease process with a wide spectrum of manifestations including pericarditis, myocarditis, valvular heart disease and less frequently, coronary artery vasculitis (CAV). CAV encompasses an emerging field of diseases which differ from conventional atherosclerotic disease and have a proclivity for the younger population groups. CAV portends multiple complications including the development of coronary artery aneurysms, coronary stenotic lesions, and thrombosis, all which may result in acute coronary syndromes. There are several aetiologies for CAV; with Kawasaki’s disease, Takayasu's arteritis, Polyarteritis Nodosa, and Giant-Cell Arteritis more frequently described clinically, and in literature. There is a growing role for multi-modality imaging in assisting the diagnostic process; including transthoracic echocardiography, cardiac magnetic resonance imaging, computed tomography coronary angiography, fluorodeoxyglucose-positron emission tomography and conventional coronary angiogram with intravascular ultrasound. Whilst the treatment paradigms fundamentally vary between different aetiologies, there are overlaps with pharmacological regimes in immunosuppressive agents and anti-platelet therapies. Interventional and surgical management are is a consideration in select populations groups, within a multi-disciplinary context. Further large-scale studies are required to better appropriately outline management protocols in this niche population.

## Background

Vasculitis is a general term that encompasses a group of disorders characterised by inflammation of blood vessels [1]. The majority of vasculitic disorders affect multiple organ systems and therefore can have a myriad of presentations necessitating the need for a high index of clinical suspicion. Whilst cardiac manifestations of systemic vasculitis are rarely seen in practice, their presence often serve as a poor prognostic factor [2]. Cardiac manifestations that are frequently described in literature include pericarditis, myocarditis, valvular heart disease and less commonly, coronary artery vasculitis (CAV) [3].

CAV represent an important differential diagnoses to consider, in younger patients with unexplained acute coronary syndromes (ACS) or congestive cardiac failure, especially in the setting of a known primary or secondary vasculitis [4]. These events may be principal manifestations in the younger population, who do not have traditional Framingham cardiovascular risk factors [5]. A high index of clinical suspicion is required for appropriate work-up, especially as initial investigative modalities carry variable levels of diagnostic sensitivity or specificity.

The most frequently described aetiologies of CAV include Polyarteritis Nodosa (PAN), Kawasaki’s Disease (KD), Takayasu’s Arteritis (TA) and Giant Cell Arteritis (GCA) [6]. In lieu of the high morbidity and mortality associated with CAV, early diagnosis and appropriate treatment are crucial to curb the trajectory of the unique disease process. This requires integration of appropriate cardiac-specific pharmacotherapy, immunosuppressive agents and interventional therapies [2–7].

## Epidemiology

There remains a paucity of data on the global epidemiology of CAV, and this largely stems from various factors including under-diagnosis, low incidence rates and variable levels of sensitivity and specificity amongst current diagnostic tools [2]. Primary medium-vessel vasculitides such as PAN (incidence rate 4–10 per million) and KD (incidence rate 2 per million), may have coronary involvement of up to 50% and 20% respectively [2, 8] See Table 1. Furthermore, large vessel vasculitides such as TA and GCA with an estimated annual incidence of 1–2 per million and 1–3 per million respectively, also have contrasting frequencies of coronary involvement (10–30% and < 1% respectively). Other rarer diagnoses such as Erdheim Chester Disease, whilst described less than a hundred times in literature, are associated with high rates of coronary involvement (> 50% in index cases) [2, 9, 10].

**Table 1:** Summary of diagnostic findings in coronary artery vasculitis

Coronary vasculitis	Frequency of coronary artery involvement [2] (%)	Histopathologic findings [19]	Coronary angiography findings [2]	Epicardial coronary arteritis [22]	Coronary microvascular disease [22]	Intracavitary thrombus [22]
Polyarteritis Nodosa	10–50	Pan-arteritis with intramural, perivascular lymphocytes and macrophage infiltrates	Aneurysm and narrowing alternating	+	+	–
Kawasaki’s disease	20	Multi-cellular infiltrate with necrosis of internal elastic lamina	Large coronary aneurysms	+	+	+
Takayasu arteritis	10–30	Hyperplasia and granulomatous arteritis	Ostial stenosis Skip lesions	+	–	+
Giant cell arteritis	< 1	Transmural mononuclear cell infiltrate in the internal elastic lamina	Smooth narrowing	+	–	–

(+) = present; (–) = not present

## Clinical presentation

The clinical presentation of CAV is complex, reflective of its unique underlying pathophysiology. The presentation of CAV may manifest with typical angina, acute myocardial infarction (AMI), atrial and ventricular arrhythmias, conduction disturbances or cardiac failure [3]. The pathogenesis that belies atherosclerotic coronary artery disease (CAD) compared to CAV lends to different treatment approaches and thus it is crucial to identify the classic features of implicative vasculitides. For example, patients with TA have a high burden of constitutional symptoms, limb claudication and cerebrovascular involvement [11]. Contrastingly in GCA, patients will often have concomitant headaches, jaw claudication and acute visual loss [2]. In KD, patients often have associated conjunctivitis, lymphadenopathy, rash and hyperaemia of the lips and extremities [12]. Finally, in PAN, patients can typically present with abdominal pain, livedo reticularis or peripheral neuropathy [2]. It is therefore extremely important to perform a complete multi-organ assessment in these patients, to decipher the underlying aetiology for targeted therapy.

## Pathogenesis and pathology

The pathogenesis of CAV is complex and involves an interplay of many host factors including immune-mediated inflammation and auto-antibody dependant processes [13]. Importantly, the overproduction of pro-inflammatory cytokines, specifically interferon-gamma (IFN-γ), Tumour necrosis factor-alpha (TNF-α) and Th-1 interleukins have been observed in such vasculitic processes [14]. Each vasculitis presents with varying histopathological findings on biopsy. PAN associated CAV presents with pan-arteritis with intramural, perivascular lymphocyte and macrophage infiltration, with a resultant destructive coronary vasculitis and classic fibrinoid necrosis [15]. TA and GCA patients show evidence of intimal hyperplasia, granulomatous arteritis, and coronary atherosclerosis. In patients with KD, there is an infiltration of the arterial wall with a multi-cellular infiltrate with subsequent necrosis of the internal elastic lamina [15, 16]. Whilst the phenotypic result is frequently similar amongst these pathologies, the underlying pathogenesis is different and requires consideration of varying therapeutic approaches. As such, chronic inflammation results in scar tissue formation, necrosis and may result in coronary artery aneurysmal (CAA) formation [14]. CAA’s are a rare entity that occurs secondary to localised dilatation of a coronary artery from vessel wall weakening [17]. The underlying pathology stems from overactivity of the metalloproteinases and metalloelastases which primarily degrade elastin and collagen [18]. Arterial Thrombosess (i.e. coronary artery thrombosis) may also occur in CAV, possibly leading to vascular occlusion and consequently myocardial oschaemia, for instance in KD, where CAA formation may predispose to this mechanism [19, 20]. CAV also increases predisposition to typical CAD with resultant myocardial ischaemia, through atherosclerotic inflammatory changes [21]. There are several mechanisms involved in this process; namely coronary artery inflammation and extension from adjacent aortitis [22]. Furthermore, the development of coronary artery embolism from aortic valvulitis, complicating CAV, has also been described in the literature [23].

## Diagnostics

Prompt diagnosis and early institution of treatment lead to improved patient outcomes. This is of key importance as a large proportion of cardiac manifestations of vasculitis can be clinically silent during the early stages of the disease process, along with logistical difficulty in conducting a coronary artery biopsy for histo-pathological assessment [24]. Important laboratory investigations, other than baseline laboratory investigations, include elevated inflammatory markers such as C-reactive protein (CRP), Erythrocyte Sedimentation Rate (ESR), high sensitivity troponin assay and an extended vasculitis panel [25]. In addition to laboratory investigations, multi-modality imaging has a growing role in the diagnostics of CAV, despite their limitations. These investigations should be performed in patients who present with acute coronary syndrome on the background of known or suspected vasculitis.

### Echocardiography

**Fig. 1:**
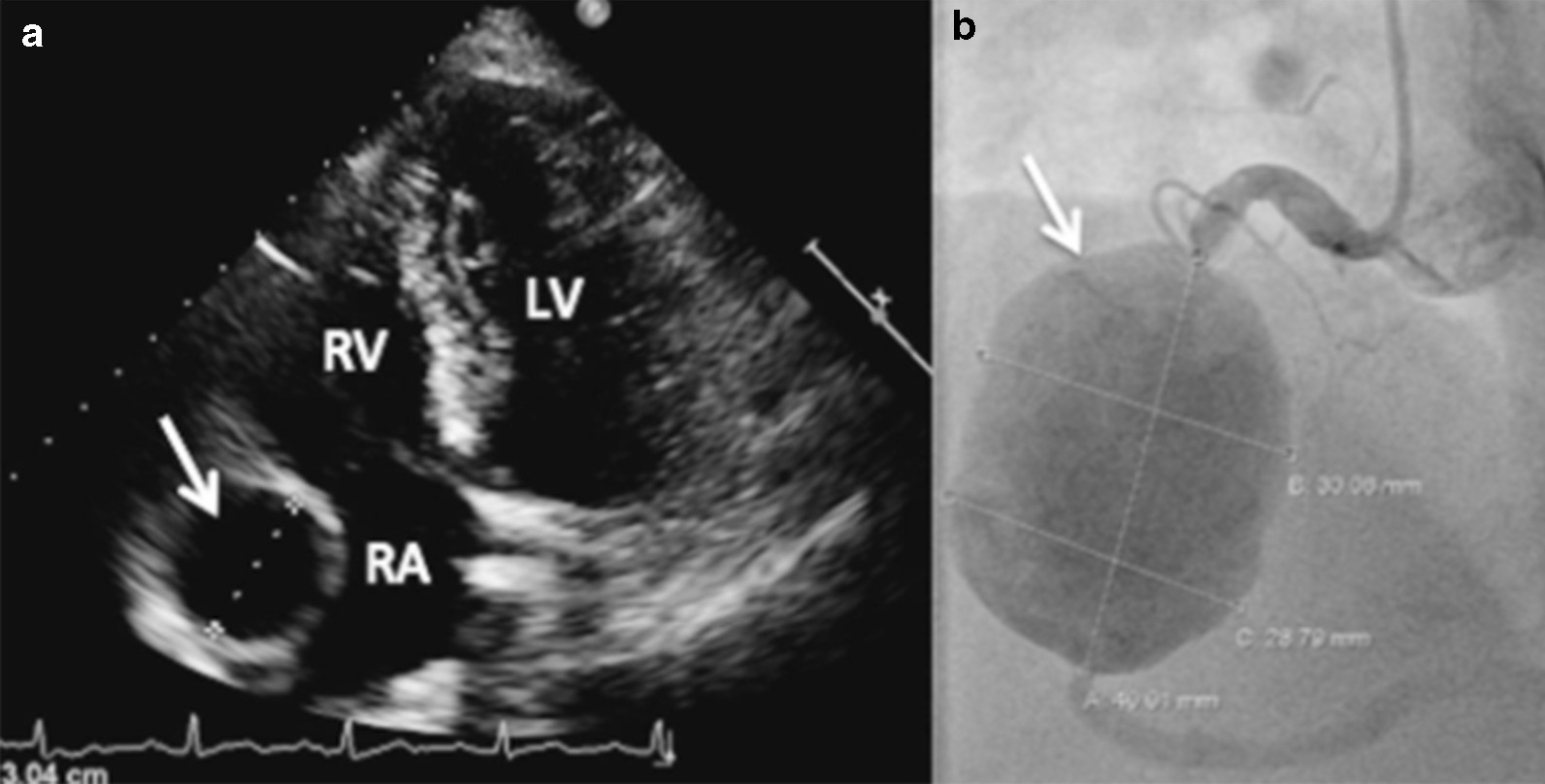
Echocardiogram shows a 30 mm wide hypoechoic structure lateral to the right atrium (arrow) (**a**). The structure is diagnosed as 30 × 40 mm right coronary aneurysm (arrow) using coronary angiography (**b**). (Adapted with permission from Ebersberger U, Rieber J, Wellmann P, Goebel C, Gansera B. Polyarteritis nodosa causing a vast coronary artery aneurysm. J Am Coll Cardiol. 2015 10;65(5):e1–2. https://doi.org/10.1016/j.jacc.2013.08.1667. PMID: 25660937) Copyright [2013] by Ullrich Ebersberger, JACC

The most readily available, non-invasive imaging modality, is transthoracic echocardiography (TTE) and is largely used as the initial investigative tool [26]. TTE is the most accessible tool for both functional and structural assessment of the heart in systole and diastole and should be performed at baseline and upon follow-up post-treatment. TTE also has reasonable sensitivity in detection of several abnormalities in the Left Main Coronary Artery (LMCA) and Right Coronary Artery (RCA) region, including aneurysmal dilatation and thrombi formation [26]. See Fig. 1. There has been an emergence of advanced techniques in echocardiography, such as Speckle Tracking Echocardiography (STE) which is able to assess cardiac function via myocardial deformation parameters including strain, strain rate and rotation. Several inflammatory conditions have been found to have evidence of myocardial involvement detected by STE [27]. For instance, in the context of KD, a study by Yu et al. showed that in comparison to controls, those with coronary artery complications had worse left ventricular dynamics, including STE [28], suggestive that STE can add value in the assessment and detection of cardiac dysfunction, and perhaps suggest coronary artery disease, in KD. The main limitations of TTE include patient body habitus, operator technique expertise, and low sensitivity of detection of coronary artery involvement in vasculitis [26–28].

### Computed tomographic coronary angiography (CTCA)

There is a growing role for routine CTCA in diagnostics, prognostication and treatment planning for patients with CAV [29]. CTCA is also a rapidly emerging imaging modality for coronary arteries as it highlights dilatations, aneurysms and stenotic lesions with higher temporal resolution and motion-free images [30]. CTCA can also provide three-dimensional reconstructions for the evaluation of complex anatomical structures and hence can be of aid in different vasculitic pathologies [31]. Furthermore, CTCA is able to provide information on disease progression and chronicity, for example, patients with detectable coronary artery involvement on CTCA were found to have a significantly higher median vasculitic disease duration (176 months vs. 21 months, *p* = 0.013) [32]. These findings highlight the growing role of CTCA as a non-invasive diagnostic tool in these patients with significant structural abnormalities. Despite this, the limitations of CTCA include contraindications in renal failure (high predisposition in vasculitis), high false negatives in the setting of heavy coronary artery calcification and inherent lower spatial resolution of epicardial vessels in comparison to invasive coronary angiography [29–32].

### Cardiac magnetic resonance imaging (CMR)

Cardiac magnetic resonance imaging (CMR) is a non-invasive imaging modality with no ionizing radiation (important in childhood KD), higher resolution, and visualisation of cardiac structures when compared to CTCA and TTE respectively. The most important advantage of CMR is the ability to visualise lumen and vessel walls with high resolution in order to assess vessel wall thickening and disease activity [80]. Specifically, in the diagnostics of CAA and arterial involvement, contrast CMR angiography and time-of-flight angiography have been shown to be non-inferior to conventional coronary angiography [33]. Additionally, free-breathing three-dimensional coronary magnetic resonance angiogram is also a non-invasive modality, and has shown benefit for follow-up in patients with KD with presence of arterial involvement. MRA is known to have an approximate sensitivity of 100% in diagnosis of CAV and its specific complications such as CAA, stenotic lesions and occlusions, making it a useful add on to CMR [33, 34]. The limitations of CMR include cost, availability, and contra-indications in the setting of cardiac devices and renal failure [33–35].

### Fluorodeoxyglucose-positron emission tomography (FDG-PET)

There is a growing role for the use of FDG-PET in the diagnostic evaluation and management of patients with systemic vasculitis [36]. FDG is fundamentally a glucose analogue and tends to accumulate in metabolic “hot spots” such as in inflammatory and autoimmune processes [37]. It has a high sensitivity rate of 77–92% and a specificity of 89–100% for the detection of large-vessel cardiac vasculitis [38]. Coronary vasculitis may first be suspected when patients with pyrexia of unknown origin or constitutional symptoms undergo FDG-PET scanning as part of their investigative work-up and the finding of areas of increased metabolic activity in coronary vessels with a circumferential pattern may be identified, suggestive of active coronary artery vasculitic disease [39]. Furthermore, FDG PET can also be used to monitor for signs of disease activity and may also be used for assessment of response to therapy, particularly given these changes may preceed anatomical changes that are detected by other imaging modalities such as CT or MRI [39]. In contrast, FDG-PET may not be useful in differentiating vasculitis from atherosclerotic disease, which may appear in a spectrum of findings, including, focal increased uptake, mild diffuse uptake, or no uptake, and hence physicians may require additional investigations into atherosclerotic disease to differentiate it from vasculitis [39]. Therefore, while FDG-PET may be useful in predicting potential vasculitc coronary artery involvement, specifically in higher-risk patients with cardiac vasculitis, it does not always discriminate between coronary atherosclerosis, which is more common than coronary artery vasculitis. The important limitations of FDG-PET include low availability, inability to differentiate between disease processes and false negatives in the setting of immunosuppressive therapy [36–39].

### Coronary angiography and intravascular ultrasound (IVUS)

Coronary Angiography is an important diagnostic and management tool as it provides information on the location of CAV stenosis and thrombosis, with the ability to provide therapeutic benefits through angioplasty and stent. Furthermore, it can characterise CAA locations and differentiate plaque types and thrombotic occlusions [40]. See Fig. 1. IVUS during angiography is considered the gold standard for evaluation of the integrity of arterial wall structure [41]. IVUS can be used as an adjunct to assess the structure and function of the vessel wall and hence is an invaluable tool in the overall diagnostics process [41, 42]. The limitations of coronary angiography include contraindications to invasive testing, renal failure, and radiographic factors such as distortion of images and inappropriate stenosis measurement [41, 42].

## Management

**Fig. 2:**
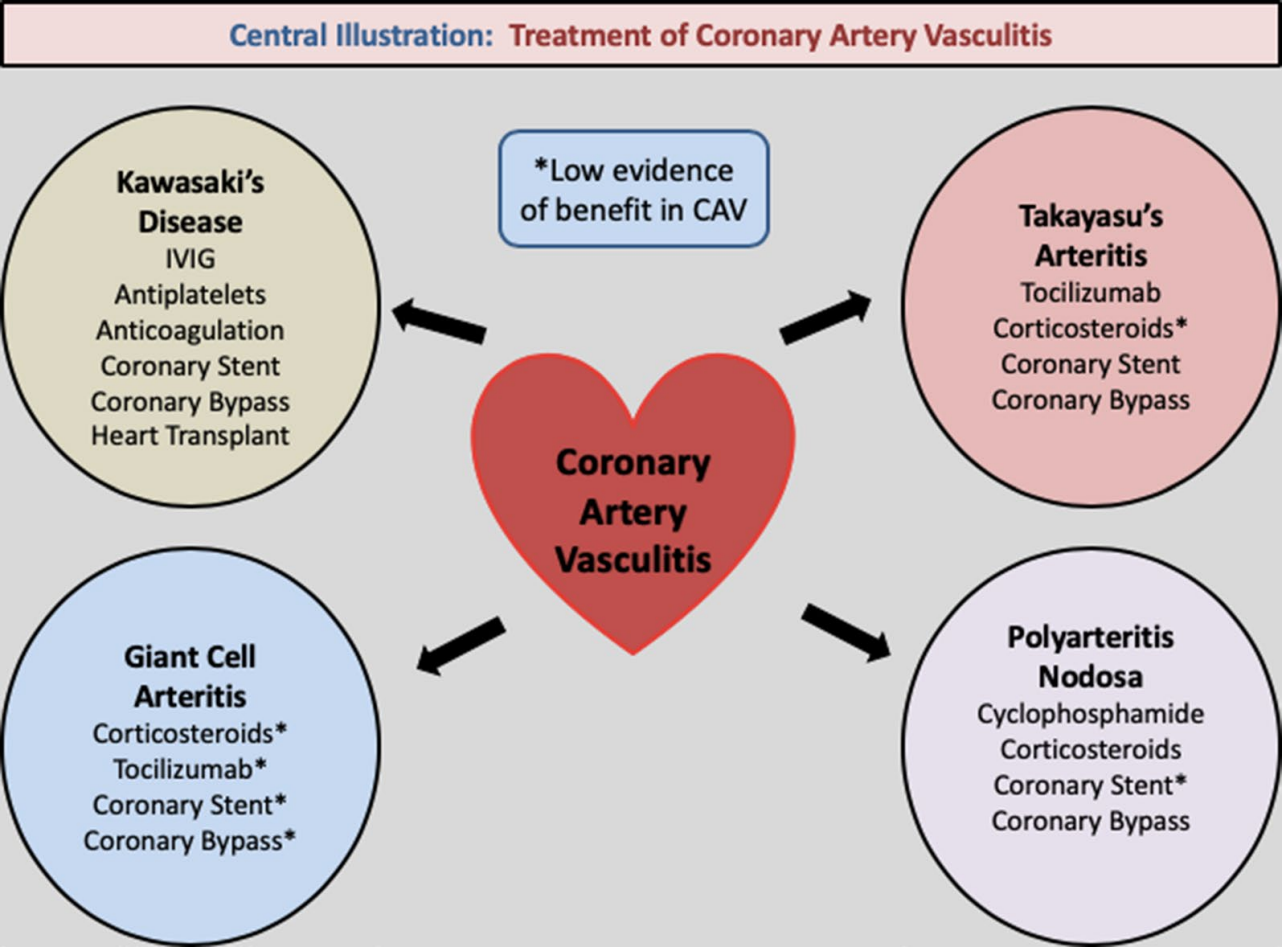
Central illustration: treatment of coronary artery vasculitis. IVIG, Intravenous Immunoglobulin; CAV, Coronary Artery Vasculitis

The management of CAV is largely predicated on accurate diagnostics and the identification of the implicative pathology. The decision on appropriate treatment is dependent on the degree of cardiac involvement, namely of the myocardium, epicardium, endocardium and conducting system [8]. The majority of current literature has focused on four disease processes; namely KD, TA, PAN, and GCA. See Fig. 2: Illustration.

### Kawasaki disease

KD forms the majority of CAV, seen with a higher degree of focus in current literature. KD is a predominant vasculitis of childhood that primarily affects the coronary arteries (~ 25% of patients), and is the most common cause for CAA [43]. Studies indicate the right coronary artery is most commonly affected (~ 35%) followed by the left anterior descending (~ 32%) [44]. In the acute phase of KD, Intravenous Immunoglobulin (IVIG), corticosteroids and aspirin monotherapy have revolutionised treatment, forming the cornerstone of most management paradigms, with a significant improvement in overall patient outcomes [12]. Furthermore, a single centre study has shown that coronary artery abnormalities on initial transthoracic echocardiogram (TTE) have a high sensitivity (~ 81%) in predicting non-response to IVIG (~ 20% of KD population). As such, serial TTE is routinely performed at baseline and at 6 weeks post-treatment to re-assess the coronary arteries for treatment failure [12]. Other identified factors associated with non-response to IVIG in KD include low albumin (< 3.3 g/L, *p* < 0.001), low haemoglobin (< 10.2 g/dL, *p* < 0.001), low sodium (< 136 meq/L, *p* < 0.001) and high procalcitonin (≥ 5.5 ng/ml, *p* < 0.001) [45]. Therefore, routine laboratory investigations and echocardiography should be performed at baseline for prognostication and to guide therapeutics.

Importantly, IVIG with concomittant steroid therapy has shown to significantly reduce the risk of coronary complications (OR: 0.3; 95% CI 0.20–0.46) with similar results found in patients with a high risk of IVIG resistance (OR: 0.2; 95% CI 0.1–0.36) in a recent meta-analysis [46]. However, these studies were largely performed in Japan, with evidence that Japanese scoring systems for IVIG resistance and aneurysms have lower sensitivity when analysed with the other populations globally [47, 48].

There is currently no established management protocol for optimal management of CAA in KD, with current treatment paradigms based on smaller retrospective analyses with respect to pharmacotherapy, percutaneous and surgical approaches. In the older population group with KD associated CAA, there is an increased focus on risk factor modification aimed at mitigating cardio-metabolic burden [39]. Despite this, the role of dual antiplatelet (DAPT), therapeutic anticoagulation and pharmacotherapeutics with angiotensin-converting enzyme inhibition has not yet been completely established for this population group [44, 49, 50].

Interventional approaches to the management of KD include percutaneous coronary intervention (PCI) to restore blood flow in CAA, with current American Heart Association guidelines recommending PCI in those with either a single vessel involvement or focal multi-vessel disease [44, 47]. In these patients, despite current recommendations of covered stents, there is a risk of restenosis and thrombosis [51, 52]. Furthermore, late angiographic evaluation after drug-eluting stent implantation has a higher risk of CAA development (1.25% of patients, 95% CI 0.58–1.93) [53]. Development of CAA post-DES implantation has been associated with higher Major Adverse Cardiac Events (MACE) (26.9% vs. 2.2%, *p* < 0.001) and nonfatal MI (11.5% vs. 0%, *p* < 0.001) when compared to patients without CAA development post-DES implantation [54]. Surgery also remains an option for patients with obstructive coronary disease and there is a consideration for aneurysmal resection/thrombectomy in the presence or absence of bypass grafting [51, 55, 56].

In patients with large CAA, with an internal luminal diameter *Z* score ≥ 10 or absolute dimension ≥ 8 mm, therapeutic anticoagulation can be considered in addition to antiplatelet therapy [57]. Sugahara et al. have shown that in patients with KD with giant CAAs, the incidence of AMI was significantly lower in the combination therapy group of aspirin and warfarin than in patients treated with aspirin alone (1 patient vs. 16 patients, *p* < 0.05) [57]. The benefit of therapeutic anticoagulation (> 60% in therapeutic time) seen in large CAA’s is mirrored in coronary artery ectasias (CAE) with less composite MACE events, non-fatal MI and cardiac death (*p* = 0.03) compared to patients without therapeutic anticoagulation [58]. Long-term management is primarily focused on the prevention of thrombosis and myocardial ischemia [47]. In patients with lower risk (normal coronary arteries on screening or transient dilatation), cardiovascular risk factor reduction is given higher importance [59]. In patients who are at higher risk (with medium-large vessel CAAs), it is important to consider periodic monitoring with serial electrocardiograms and TTE [47, 59].

Coronary Artery Bypass Grafting (CABG) and cardiac transplantation remain surgical options for KD populations with a higher burden of disease. KD who have undergone CABG, cardiac event-free rates at 25 years have been approximately 60% (95% CI 46–72). Furthermore, 20-year graft patency rate was approximately 87% (95% CI 78–93) for arterial graft versus 44% (95% CI; 26–61) for venous grafts (*p* < 0.001). Similar findings were seen in patients with non–left anterior descending artery lesions, highlighting the importance of arterial grafts for long-term disease-free survival [60].

Cardiac transplantation may be considered in patients deemed not suitable for revascularisation; namely in the presence of distal coronary stenosis, multi-vessel CAA's or those who have irreversible myocardial damage, with resultant severe LV systolic failure, incessant ventricular arrhythmias and prior cardiac arrest [61, 62]. KD patients who have undergone cardiac transplantation have shown good outcomes with a 90% survival rate upon 6-month follow data [62]. In the context of monitoring KD disease activity with conventional inflammatory biochemical markers, it is worth noting that such markers are nonspecific and may reflect inflammatory processes from other aetiologies; however, there have been associations [63], such as the finding that elevated procalcitonin levels may be useful in predicting those who may be have an increased risk for IVIG-resistant disease [64, 65].

### Takayasu’s arteritis

The three most common lesions identified in TA are stenosis or occlusion of the coronary ostia, diffuse or focal coronary arteritis, and CAAs. These findings are detected in up to 60% of patients on coronary angiography (< 20% symptomatic cases) [3, 66]. Systemic therapy with corticosteroids along with other immunosuppressive agents is indicated when the cause of CAD is presumed to be vasculitic [3]. There is also growing evidence for more novel agents such as Tocilizumab (anti-IL6) in the restoration of coronary artery function in select patient groups [67]. In patients with TA with coronary involvement, the use of Tocilizumab has been associated with a reduction in CRP (*p* = 0.006), ESR (*p* = 0.011) and Kerr-score (*p* = 0.007) for 6 months post-treatment [67]. Importantly, this treatment reduced the total number of coronary artery lesions from 23 to 15 with an improvement in vascular wall thickening [67]. These findings highlight the benefits of directed therapy in TA populations.

CABG and balloon angioplasty followed by stenting are considerable options in patients with a larger number of coronary artery lesions [2, 3]. CABG may not be preferred during periods of active vasculitis but does have the added benefit of less in-stent restenosis than angioplasty with stenting (mean 45% vs. 22.5%) [68, 69]. This risk of in-stent restenosis may not necessarily be reduced by the use of drug-eluting stents (DES) and thus CABG may be considered during periods of quiescent vasculitis, where indicated [70]. Soeiro et al. describe a case with DES to the LMCA who presented 6 months post-insertion of the stent with 90% in-stent restenosis in the context of minimal inflammatory activity (CRP 2 mg/L and ESR 16 mm/h) [70]. These findings are reflective of the possibility of surrounding vasculilitis in the aortic and subclavian arteries that may complicate revascularisation procedures [70]. Monitoring of disease activity in TA requires assessment of patient clinical symptoms, physical examination, biochemical markers and imaging. While acute phase reactants, such as CRP and ESR may be used for disease monitoring, they are nonspecific and may even be normal during active disease states [71].

### Polyarteritis nodosa

The management of PAN associated with CAV requires a structured approach given high rates of coronary involvement (~ 50%) [2]. The goal of treatment in PAN associated CAV is to reduce overall vascular inflammatory burden, which may induce disease remission in coronary arteries. Cyclophosphamide can be used in addition to corticosteroids in patients with moderate to severe PAN, especially those who may present with an ACS or CAAs [72]. In this patient group, 12-monthly pulses of cyclophosphamide have resulted in a significantly lower relapse rate (HR: 0.34, *p* = 0.02) and a higher survival rate at 32 months (HR: 0.44, *p* = 0.02) [72, 73]. Despite aggressive immunosuppression, there is often a requirement for revascularisation as well as consideration of CABG depending on the severity of PAN [3]. Pre-CABG work-up is crucial with angiographic assessessment of the internal thoracic artery for evidence of stenosis or occlusion, as these have been found to complicate revascularisation procedures [74, 75].

### Giant cell arteritis

The prevalence of coronary artery involvement is rarer in GCA than its counterparts described above, with the majority of CAV found in autopsy findings [76]. Concurrently, results from a GCA cohort study (n = 3408) have shown the incidence AMI was 10 versus 4.9 per 1000-person years, when compared to patients without GCA. These findings were even more prominent in the first month after GCA diagnosis (HR for AMI: 11.89, 95% CI 2.40–59.00), hence signifying the burden of vascular disease in this group [77]. Tocilizumab has been shown to be effective for the treatment of GCA in addition to corticosteroids (relapse-free survival achieved in 85% of patients versus 20% with corticosteroid monotherapy) (*p* = 0.001), as demonstrated in a small case series [78, 79]. Trials to date have demonstrated the efficacy of tocilizumab in GCA, without assessment of effects on CAV. Given the temporal relationship of GCA with TA, these benefits are assumed in this population group. Despite these findings, patients with GCA who present with AMI are less likely to undergo interventional therapies when compared to non-GCA counterparts (19% vs. 50%, *p* = 0.015), highlighting the higher overall disease burden [19]. In the context of biochemical monitoring, CRP and ESR are useful in the assessment of disease activity improvement, where these measures often decrease following pharmacological or interventional therapies, where CRP decreases more rapidly in contrast to ESR [80]. Such is the important role of these biomarkers that the decision to taper glucocorticoid therapy is often predicated on decreases in ESR and CRP, although it is important to be cognisant of the caveats of such biomarkers, given that they increase in other causes of inflammation.

## Conclusions

While CAV and its sequelae portend a poor prognosis, prompt diagnosis and early institution of therapy can result in higher survival rates. The role of multi-modality imaging as a non-invasive diagnostic tool is pivotal in this disease entity, and its role in conjunction with emerging medical, interventional and surgical therapies continues to grow. Further larger-scale studies are required to deduce the optimal approach for this niche population, thereby facilitating more concrete treatment paradigms to improve patient outcomes.

## Data Availability

No data required for this review. All authors happy for materials in this publication to be re-used.
